# Small Cell Lung Carcinoma Cells Depend on KIF11 for Survival

**DOI:** 10.3390/ijms25137230

**Published:** 2024-06-30

**Authors:** Yuji Sakuma, Sachie Hirai, Miki Yamaguchi, Masashi Idogawa

**Affiliations:** 1 Department of Molecular Medicine, Research Institute for Immunology, Sapporo Medical University School of Medicine, Sapporo 060-8556, Japan; hirai@sapmed.ac.jp (S.H.); mikiyama@sapmed.ac.jp (M.Y.); 2 Department of Medical Genome Sciences, Cancer Research Institute, Sapporo Medical University School of Medicine, Sapporo 060-8556, Japan; idogawa@sapmed.ac.jp

**Keywords:** small cell lung cancer (SCLC), kinesin family member 11 (KIF11), BCL2-like 1 (BCL2L1), apoptosis

## Abstract

Few efficacious treatment options are available for patients with small cell lung carcinoma (SCLC), indicating the need to develop novel therapeutic approaches. In this study, we explored kinesin family member 11 (KIF11), a potential therapeutic target in SCLC. An analysis of publicly available data suggested that *KIF11* mRNA expression levels are significantly higher in SCLC tissues than in normal lung tissues. When KIF11 was targeted by RNA interference or a small-molecule inhibitor (SB743921) in two SCLC cell lines, Lu-135 and NCI-H69, cell cycle progression was arrested at the G2/M phase with complete growth suppression. Further work suggested that the two cell lines were more significantly affected when both KIF11 and BCL2L1, an anti-apoptotic BCL2 family member, were inhibited. This dual inhibition resulted in markedly decreased cell viability. These findings collectively indicate that SCLC cells are critically dependent on KIF11 activity for survival and/or proliferation, as well as that KIF11 inhibition could be a new strategy for SCLC treatment.

## 1. Introduction

Over the past two decades, highly effective treatments have been developed for patients with non-small cell lung carcinoma (NSCLC), including tyrosine kinase inhibitors against tumors with a driver mutation and immune checkpoint inhibitors against those lacking such a genetic alteration. Accordingly, the overall survival of patients with NSCLC has been dramatically improved [1]. In contrast, little progress has been made on the development of revolutionary treatments for small cell lung carcinoma (SCLC) [2]. 

In this study, we focused on kinesin family member 11 (KIF11), also termed kinesin spindle protein or EG5. KIF11 is a motor protein involved in the formation of the bipolar spindle, with potent and specific KIF11 inhibitors having already been developed such as Arry-520, Ispinesib, and SB743921 [3,4]. Moreover, several KIF11 inhibitors entered clinical trials targeting multiple myeloma, acute myeloid leukemia, NSCLC, breast cancer, ovarian cancer, etc. [3,4]. We have recently found that high *KIF11* mRNA expression levels are significantly associated with an unfavorable outcome in patients with lung adenocarcinoma. Additionally, lung adenocarcinoma cells highly depend on KIF11 for growth [5]. However, the roles of KIF11 in SCLC remain unclear. SCLC has been considered to consist of four subtypes—SCLC-A (ASCL1 dominant), -N (NEUROD1 dominant), -P (POU2F3 dominant), and -Y (YAP1 dominant) subtypes [6]. 

This report demonstrates that KIF11 is highly expressed in SCLC tissues compared with normal lung tissues. Furthermore, KIF11 inhibition could completely suppress the growth of classic (neuroendocrine) SCLC cells, namely the SCLC-A and SCLC-N subtypes, which account for ~85% of SCLC cases [6]. SCLC cells treated with a KIF11 inhibitor displayed greater levels of apoptosis when BCL2-like 1 (BCL2L1), an anti-apoptotic BCL2 family member [7], was also inhibited. 

## 2. Results 

### 2.1. SCLC Tissues Highly Express KIF11 mRNA

**Figure 1:**
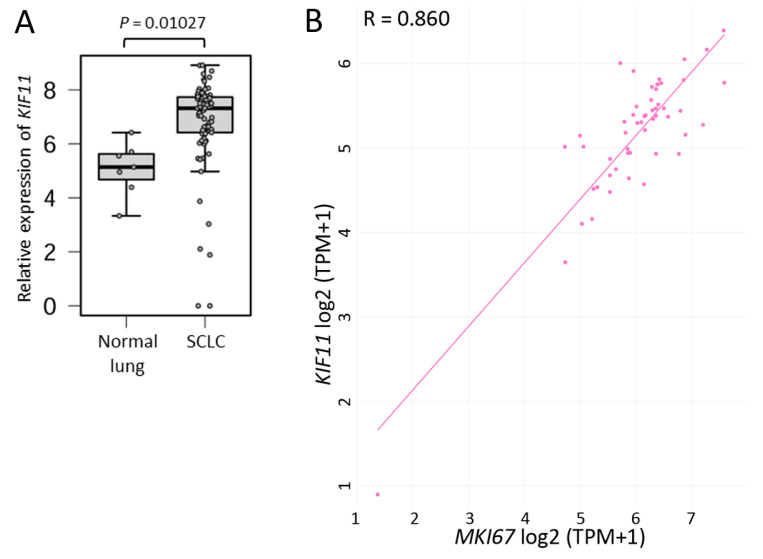
Small cell lung carcinoma (SCLC) tissues have higher *KIF11* mRNA expression levels than normal lung tissues. (**A**) *KIF11* mRNA expression patterns in normal lung and SCLC tissues. Expression data from 7 normal lung and 79 SCLC tissues were obtained from a previous report [9]. (**B**) Correlation between *KIF11* and *MKI67* mRNA expression levels in 53 SCLC cell lines using data from the Cancer Dependency Map [10]. Pearson’s correlation coefficient (R) = 0.860.

Figure 1A shows that *KIF11* mRNA was much more highly expressed in SCLC tissues than in normal lung tissues. In addition, the expression levels of *KIF11* mRNA were almost perfectly correlated with those of *MKI67* mRNA, a reliable cell proliferation marker [8], in SCLC cell lines (Figure 1B). These findings, at least in part, led us to hypothesize that KIF11 could be a potential therapeutic target for SCLC. 

### 2.2. KIF11 Inhibition Causes Cell Cycle Arrest at the G2/M Phase in Lu-135 and H69 Cells

**Figure 2:**
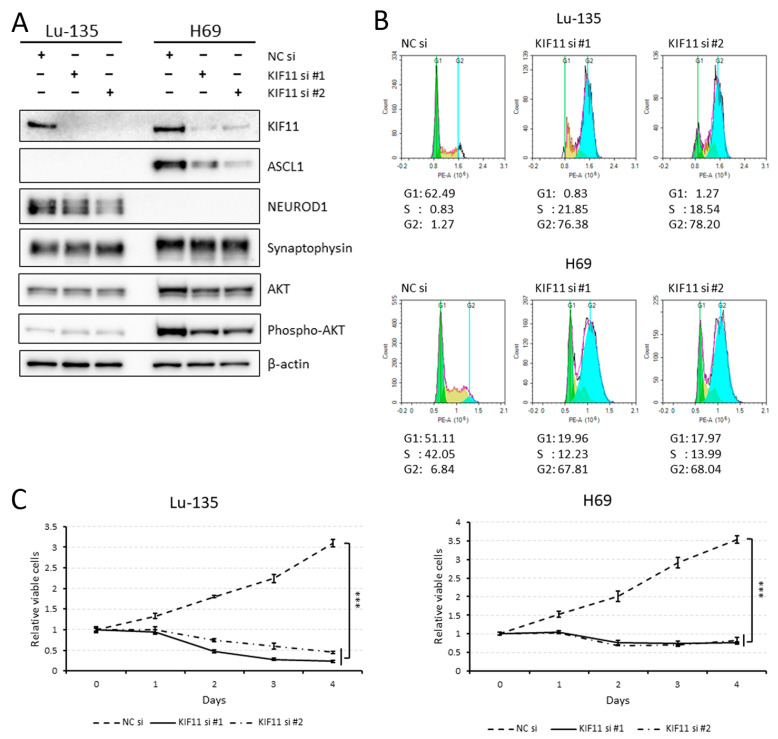
KIF11 knockdown with small interfering RNAs (siRNAs) elicits cell cycle arrest at the G2/M phase in Lu-135 and H69 cells. (**A**) The Western blot analysis of KIF11, ASCL1, NEUROD1, synaptophysin, AKT, phospho-AKT, and β-actin protein expression in Lu-135 and H69 cells. Cells were reverse-transfected with a negative control (NC) siRNA, KIF11 siRNA #1, or KIF11 siRNA #2 (10 nM each) and cultured for 48 h. (**B**) The cell cycle analysis of the two SCLC cell lines using flow cytometry. The cells were treated as described in (**A**). (**C**) The effects of KIF11 knockdown on the viability of Lu-135 and H69 cells. Cells were reverse-transfected with NC siRNA, KIF11 siRNA #1, or KIF11 siRNA #2 (10 nM each) and cultured for 24 h. Cells (3 × 10^3^) were then seeded in 96-well plates and cultured for another 96 h. Cell viability at days 0, 1, 2, 3, and 4 was assessed in triplicate. The data are presented as the mean ± standard deviation (SD). *** *p* < 0.001 (at day 4).

As shown in Figure 2A, Lu-135 and H69 cell lines were classified as SCLC-N and SCLC-A, respectively, based on the current SCLC molecular subtypes [6]. Lu-135 or H69 cells with either KIF11 expression knocked down by RNAi or treatment with the KIF11 inhibitor SB743921 [3,4] were found to have cell cycle arrest at the G2/M phase (Figure 2B; Supplementary Figure S1). Although KIF11 knockdown completely suppressed the growth of Lu-135 and H69 cells, the AKT remained phosphorylated when KIF11 was silenced (Figure 2A,C), suggesting that KIF11 does not directly affect the PI3K/AKT pathway. 

### 2.3. Another KIF11 Inhibitor, Ispinesib, Is More Effective against SCLC Cell Lines Expressing Higher KIF11 mRNA

As presented in Supplementary Figure S2, SCLC cell lines expressing higher *KIF11* mRNA tended to be more severely affected by Ispinesib treatment. This, together with the high *KIF11* mRNA expression in SCLC tissues (Figure 1A), suggests that most SCLC tumors would be sensitive to KIF11 inhibitors, resulting in decreased viability.

### 2.4. Lu-135 Cells Treated with SB743921 Are More Affected by BCL2L1 Inhibition

**Figure 3:**
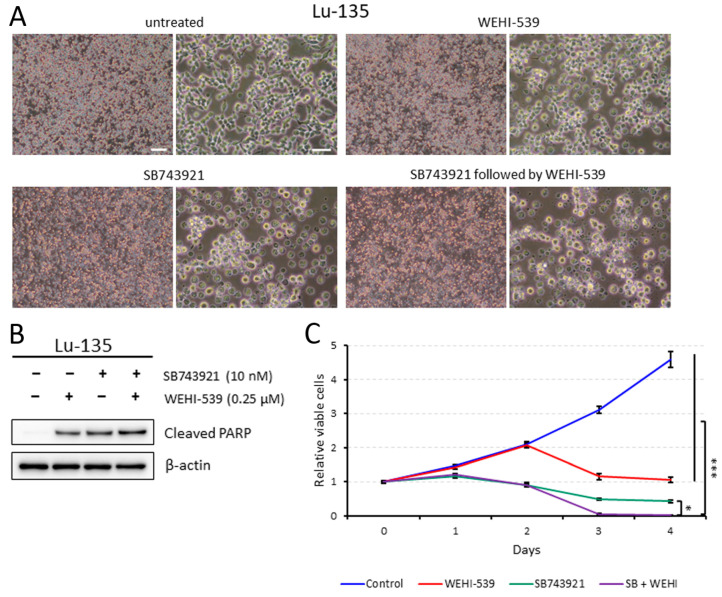
Sequential treatment of Lu-135 cells with SB743921 followed by WEHI-539 markedly decreases cell viability. (**A**) Phase contrast images of Lu-135 cells treated with WEHI-539 alone, SB743921 alone, a combination of SB743921 and WEHI-539, or neither. Cells were untreated or treated with SB743921 (10 nM) for 48 h, and then were untreated or treated with WEHI-539 (0.25 μM) for another 24 h. Scale bars = 200 µm (left; low magnification) and 50 µm (right; high magnification). (**B**) The Western blot analysis of the cells treated as described in (**A**). (**C**) The cell viability analysis of the treated Lu-135 cells. Cells were untreated or treated with SB743921 (10 nM) for 48 h, and then were untreated or treated with WEHI-539 (0.25 μM) for another 48 h. Cell viability at days 0, 1, 2, 3, and 4 was assessed in triplicate. The data are presented as the mean ± SD. * *p* < 0.05, *** *p* < 0.001 (at day 4).

SB743921 treatment alone induced apoptosis to a certain extent in Lu-135 cells, which resulted in decreased cell viability (Figure 3A–C). Moreover, sequential therapy with SB743921 followed by the BCL2L1 inhibitor WEHI-539 [7] induced apoptosis to a little greater extent and decreased viability more significantly in Lu-135 cells (Figure 3A–C). As expected, sequential treatment with KIF11 knockdown followed by WEHI-539 had stronger effects on Lu-135 cells than KIF11 knockdown alone (Supplementary Figure S3A,B).

### 2.5. Sequentially Inhibiting KIF11 and BCL2L1 Is Also Effective against H69 Cells

**Figure 4:**
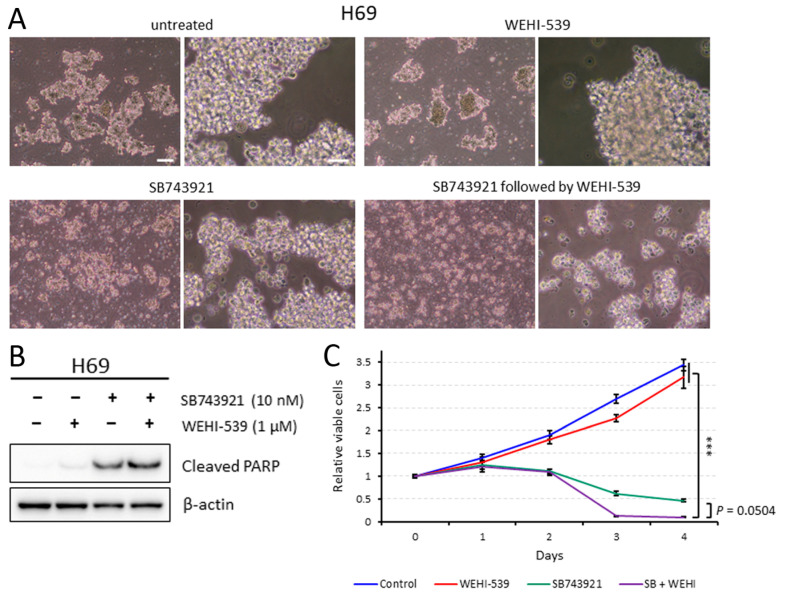
Sequential treatment with SB743921 followed by WEHI-539 is highly effective against H69 cells. (**A**) Phase contrast images of H69 cells treated with WEHI-539 alone, SB743921 alone, a combination of SB743921 and WEHI-539, or neither. Cells were untreated or treated with SB743921 (10 nM) for 48 h, and then were untreated or treated with WEHI-539 (1 μM) for another 24 h. Scale bars = 200 µm (left; low magnification) and 50 µm (right; high magnification). (**B**) The Western blot analysis of the cells treated as described in (**A**). (**C**) The cell viability analysis of the treated H69 cells. Cells were untreated or treated with SB743921 (10 nM) for 48 h, and then were untreated or treated with WEHI-539 (1 μM) for another 48 h. Cell viability at days 0, 1, 2, 3, and 4 was assessed in triplicate. The data are presented as the mean ± SD. *** *p* < 0.001 (at day 4).

Similar to in Lu-135 cells, SB743921 treatment alone induced apoptosis and reduced viability in H69 cells (Figure 4A–C). Furthermore, adding WEHI-539 enhanced the therapeutic effects of the KIF11 inhibitor (Figure 4A–C). Sequential treatment with KIF11 silencing followed by WEHI-539 also induced higher levels of apoptosis in H69 cells than KIF11 silencing alone (Supplementary Figure S4A,B).

### 2.6. Simultaneous Sirna-Mediated Knockdown of KIF11 and BCL2L1 Induces Significant Apoptosis in Lu-135 and H69 Cells

We next explored if sequential treatment with KIF11 and BCL2L1 inhibition was required to induce apoptosis and reduce viability in SCLC cells. As demonstrated in Supplementary Figures S5A–C and S6A–C, simultaneous knockdown of KIF11 and BCL2L1 similarly induced extensive apoptosis in the two SCLC cell lines examined.

## 3. Discussion

In this study, we first demonstrated that *KIF11* mRNA is highly expressed in SCLC tissues compared with normal lung tissues, with these expression levels strongly correlating with those of *MKI67* mRNA in SCLC cell lines. Knocking down KIF11 expression triggered cell cycle arrest at the G2/M phase in SCLC-A or SCLC-N cells. Additionally, the sequential or simultaneous inhibition of KIF11 together with BCL2L1 resulted in high levels of apoptosis in SCLC-A or SCLC-N cells, as well as markedly reduced viability. KIF11 has attracted great interest over the past few decades as a promising mitotic target, leading to specific small-molecule inhibitors being developed [3,4]. Although most of the KIF11 inhibitors tested in clinical trials had only limited efficacy, Arry-520 (also known as filanesib) has shown clinical efficacy in patients with multiple myeloma and has entered phase 3 clinical trials [4]. Although KIF11 was identified as a potential therapeutic target in a comprehensive study of SCLC [11], its specific roles in this disease have not been fully understood. In this study, we found that the siRNA-mediated or pharmacological inhibition of KIF11 could induce cell cycle arrest at the G2/M phase in Lu-135 and H69 cells with the induction of apoptosis. As previously observed in lung adenocarcinoma cells [5], the dual inhibition (sequential or simultaneous) of KIF11 and BCL2L1 caused significant levels of apoptosis in SCLC-A or SCLC-N cells, resulting in markedly reduced viability. Taken together, these findings suggest that inhibiting KIF11 alone and targeting both KIF11 and BCL2L1 are potential therapeutic options for patients with SCLC-A or SCLC-N.

Evidence has suggested that KIF11 inhibitor-related toxicity appears to be manageable, whereas the on-target platelet toxic effects of BCL2L1 inhibitors have been difficult to control [3,4,7]. However, there is a promising drug called DT2216, a selective BCL2L1 proteolysis targeting chimera (PROTAC) degrader. This drug consists of two ligands targeting BCL2L1 and the von Hipple–Lindau (VHL) E3 ligase, which are connected by a chemical linker. This promotes BCL2L1 ubiquitination and subsequent degradation through the ubiquitin–proteasome system [12,13]. The tumor selectivity of the PROTAC DT2216 is likely achieved from the minimal expression levels of the VHL E3 ligase in platelets, preventing them from undergoing apoptosis [12,13]. These findings led us to surmise that the dual inhibition of KIF11 plus BCL2L1 using small-molecule compounds may be feasible in a clinic. We also expect that sequential therapy with the KIF11 inhibitor followed by the BCL2L1 inhibitor could enhance the therapeutic effects compared with simultaneous inhibition. This is largely because KIF11 inhibition can induce cellular senescence while BCL2L1 inhibition can prompt senescent cells to undergo apoptosis [5,7].

Although *KIF11* mRNA is expressed at higher levels in SCLC tissues than in normal lung tissues, no SCLC tissues included in the cBioPortal database (https://www.cbioportal.org; accessed on 1 March 2024) have *KIF11* gene amplification. The specific mechanisms underlying the high KIF11 expression patterns in SCLC remain to be studied. However, high KIF11 expression levels have also been observed in small cell carcinoma of the esophagus [14]. In this study, we found an extremely high correlation between *KIF11* and *MKI67* mRNA expression levels in SCLC. Because high MKI67 expression is associated with shorter survival in patients with pulmonary neuroendocrine tumors, including SCLC [8], a high expression of KIF11 in SCLC would be compatible with its high malignancy.

This study has several limitations. Although our experimental findings suggest that the dual inhibition of KIF11 plus BCL2L1 is effective against classic (neuroendocrine) SCLC, namely SCLC-A and SCLC-N subtypes, we extensively analyzed only two SCLC cell lines in vitro. Many more SCLC cell lines need to be examined in vivo as well as in vitro. Also, it remains unsolved whether our treatment strategy has similar efficacy against the non-neuroendocrine SCLC-P or SCLC-Y subtype.

In summary, SCLC-A and SCLC-N cells critically depend on KIF11 activity for survival and proliferation. Although treatment with KIF11 inhibitors alone appears to be effective against classic SCLC, a combination approach targeting both KIF11 and BCL2L1 would be a much more potent method for fighting this disease. The dual inhibition of KIF11 and BCL2L1 is a potential new direction for SCLC therapeutic development that should be explored further.

## 4. Materials and Methods

### 4.1. Comparison of KIF11 mRNA Expression Levels in Normal Lung and SCLC Tissues

The RNA sequencing (RNA-seq) data of normal lung (*n* = 7) and SCLC (*n* = 79) tissues provided by Jiang et al. [9] was reanalyzed to elucidate *KIF11* mRNA expression levels in these tissues as described previously [15].

### 4.2. The Cancer Dependency Map

The Cancer Dependency Map (https://depmap.org/portal/; accessed on 1 March 2024) was used to evaluate to what degree *KIF11* mRNA expression levels were positively correlated with *MKI67* (marker of proliferation of Ki-67) mRNA expression levels in SCLC cell lines [10]. The database was also utilized to clarify whether the KIF11 inhibitor Ispinesib was effective against SCLC cell lines [3,4],

### 4.3. Cell Culture and Inhibitors

Two SCLC cell lines, Lu-135 and NCI-H69, were purchased from Japanese Cancer Research Resources Bank (JCRB; Osaka, Japan) and the American Type Culture Collection (ATCC; Manassas, VA, USA), respectively. The cells were maintained in an RPMI-1640 medium (FUJIFILM Wako Pure Chemical, Osaka, Japan) containing 10% (*v*/*v*) fetal bovine serum and antibiotics at 37 °C in a humidified incubator with 20% O_2_ + 5% CO_2_. We selected and used the KIF11 inhibitor SB743921 (10 nM, Selleck Chemicals, Houston, TX, USA) and the BCL2L1 inhibitor WEHI-539 (0.25 or 1 μM, Cayman Chemical, Ann Arbor, MI, USA) in this study [4,7].

### 4.4. RNA Interference (RNAi) Assay

Cells were transfected with negative control (NC) small interfering RNA (siRNA) duplexes (1027281; Qiagen, Valencia, CA, USA) or siRNA duplexes targeting KIF11 and/or BCL2L1 using the Lipofectamine RNAiMAX reagent and OPTI-MEM I (Thermo Fisher Scientific, Waltham, MA, USA) as described previously [5,15]. Two types of siRNA duplexes were purchased for transient KIF11 or BCL2L1 silencing: Silencer Select Validated siRNAs s7903 and s7904 (termed KIF11 siRNA #1 and #2, respectively; Thermo Fisher Scientific), Silencer Select Pre-Designed siRNA s224530 (termed BCL2L1 siRNA #1; Thermo Fisher Scientific), and Silencer Select Validated siRNA s1921 (termed BCL2L1 siRNA #2; Thermo Fisher Scientific). The final concentration of the siRNA used in each in vitro experiment was 10 nM. Knockdown of targeted gene expression at the protein level was verified by a Western blot analysis.

### 4.5. Western Blot Analysis

The Western blot analysis was conducted to determine protein expression levels with anti-KIF11 (clone E1L3W; 1:1000; Cell Signaling Technology (CST), Danvers, MA, USA), anti-ASCL1 (clone E5S4Q; 1:1000; CST), anti-NEUROD1 (clone D90G12; 1:1000; CST), anti-synaptophysin (clone D8F6H; 1:1000; CST), anti-AKT (clone C67E7; 1:1000; CST), anti-phospho-AKT (Ser473) (clone D9E; 1:1000; CST), anti-BCL2L1 (clone 54H6; 1:1000; CST), anti-cleaved PARP (Asp214) (clone D64E10; 1:1000; CST), and anti-β-actin (clone AC-15; 1:10,000; Sigma-Aldrich Japan, Tokyo, Japan) antibodies as previously described [5,15]. Of note, the results were based on at least three independent experiments and representative findings are shown.

### 4.6. Cell Cycle Analysis

Cells reverse-transfected with NC siRNA or KIF11 siRNA or untreated or treated with SB743921 (10 nM) were cultured for 48 h. The cells were then harvested and fixed in 75% methanol at 4 °C for 1 h. The cells were washed twice with cold phosphate-buffered saline (PBS) and stained with propidium iodide (PI) (0.5 mg/mL RNase and 0.1 mg/mL PI in PBS) for 30 min at room temperature. The stained cells were characterized using a NovoCyte Flow Cytometer (ACEA Biosciences, San Diego, CA, USA) and the DNA content was analyzed using FlowJo software version 10.

### 4.7. Assessment of Cell Viability and Apoptosis

The number of viable cells was estimated using a CellTiter Glo 3D Cell Viability Assay (Promega, Madison, WI, USA) as described previously [5,15]. All results are presented as the mean ± standard deviation (SD). Apoptosis was assessed by a Western blot analysis of cleaved PARP.

### 4.8. Statistical Analysis

A one-way analysis of variance (ANOVA) followed by the Tukey–Kramer multiple comparison test was performed to evaluate differences in cell viability between cells differentially treated in vitro. *p* < 0.05 denotes statistical significance. All statistical calculations were performed using JMP Pro software version 15 (SAS Institute Japan, Tokyo, Japan).

## Data Availability

Data are contained within the article and Supplementary Materials.

## Supplementary Materials

The following supporting information can be downloaded at: https://www.mdpi.com/article/10.3390/ijms25137230/s1.
