# Supplementary material for: Small Cell Lung Carcinoma Cells Depend on KIF11 for Survival
Source: Int J Mol Sci. 2024 Jun 30;25(13):7230. doi: 10.3390/ijms25137230 (PMC11241341; doi:10.3390/ijms25137230)
Supplement: Supplementary file 1 [file ijms-25-07230-s001.zip › ijms-3015816-supplementary.pdf]

# Small cell lung carcinoma cells depend on KIF11 for survival

Yuji Sakuma[1,*], Sachie Hirai[1], Miki Yamaguchi[1], and Masashi Idogawa[2]

[1] Department of Molecular Medicine, Research Institute for Immunology, Sapporo Medical University School of Medicine, Sapporo, Japan

[2] Department of Medical Genome Sciences, Cancer Research Institute, Sapporo Medical University School of Medicine, Sapporo, Japan

Supplementary figures

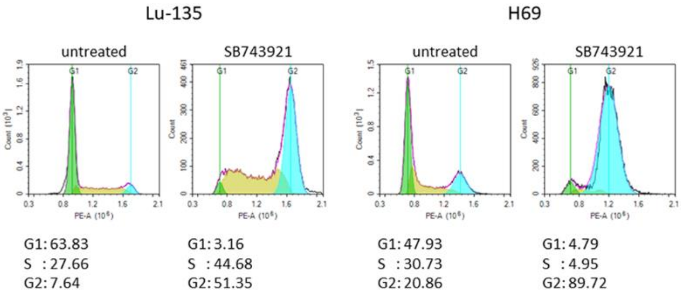


**Supplementary Figure S1.** SB743921, a KIF11 inhibitor, induces cell cycle arrest at the G2/M phase in Lu-135 and H69 cells. Cell cycle analysis of the two small cell lung carcinoma (SCLC) cell lines using flow cytometry. Cells were untreated or treated with SB743921 (10 nM) for 48 hours.

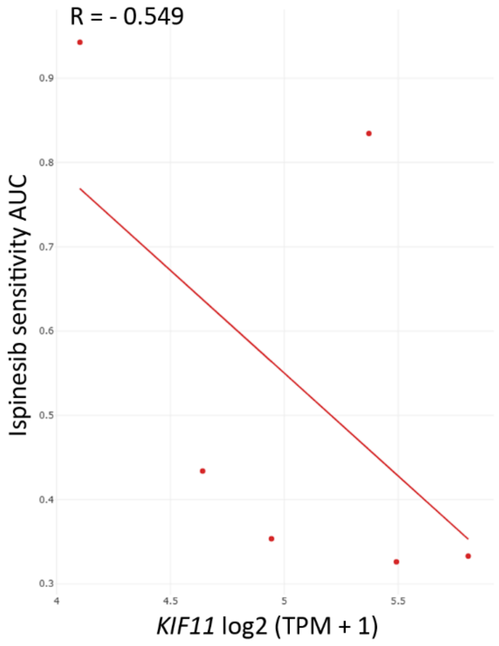


**Supplementary Figure S2.** Ispinesib, another KIF11 inhibitor, is more effective against small cell lung carcinoma (SCLC) cell lines expressing higher *KIF11* mRNA. Of note, a lower area under the curve (AUC) value indicates higher drug sensitivity. The data was obtained from the Cancer Dependency Map [15]. Pearson's correlation coefficient (R) = -0.549.

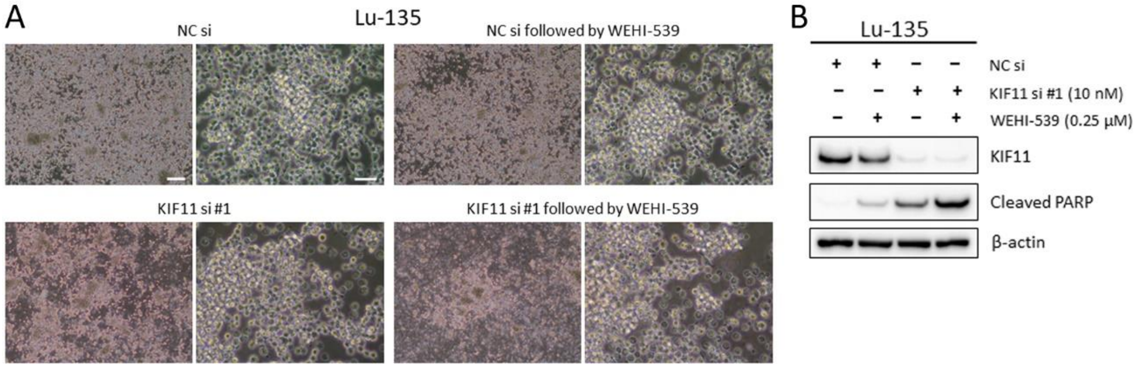


**Supplementary Figure S3.** Sequential treatment of KIF11 knockdown with small interfering RNAs (siRNA) followed by WEHI-539 induces high levels of apoptosis in Lu-135 cells. (A) Phase contrast images of Lu-135 cells treated with WEHI-539 alone, KIF11 knockdown alone, a combination of KIF11 knockdown and WEHI-539, or controls. Cells transfected with the negative control (NC) siRNA or KIF11 siRNA #1 were cultured for 48 hours, then were untreated or treated with WEHI-539 (0.25 µM) for another 24 hours. Scale bars = 200 µm (left; low magnification) and 50 µm (right; high magnification). (B) Western blot analysis of the cells treated as described in (A).

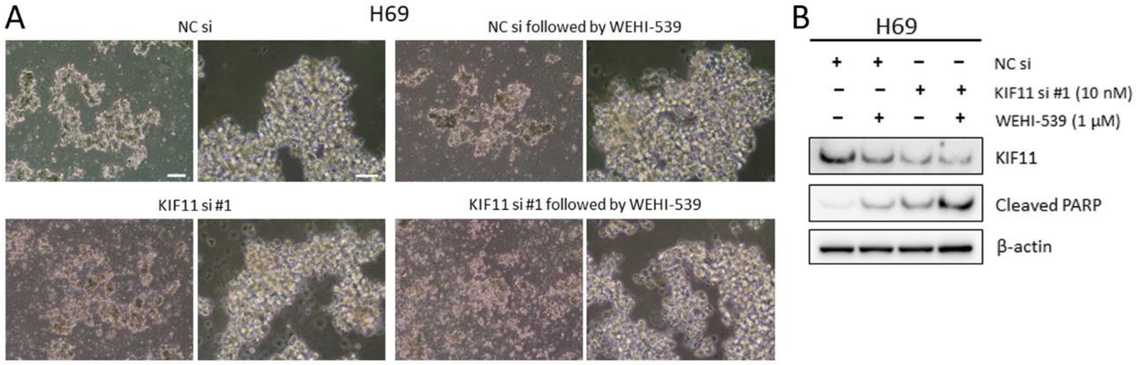


**Supplementary Figure S4.** Sequential treatment of KIF11 knockdown with small interfering RNAs (siRNAs) followed by WEHI-539 is highly effective against H69 cells. (A) Phase contrast images of H69 cells treated with WEHI-539 alone, KIF11 knockdown alone, a combination of KIF11 knockdown and WEHI-539, or controls. Cells transfected with the negative control (NC) siRNA or KIF11 siRNA #1 were cultured for 48 hours, then were untreated or treated with WEHI-539 (1 μM) for another 24 hours. Scale bars = 200 μm (left; low magnification) and 50 μm (right; high magnification). (B) Western blot analysis of the cells treated as described in (A).

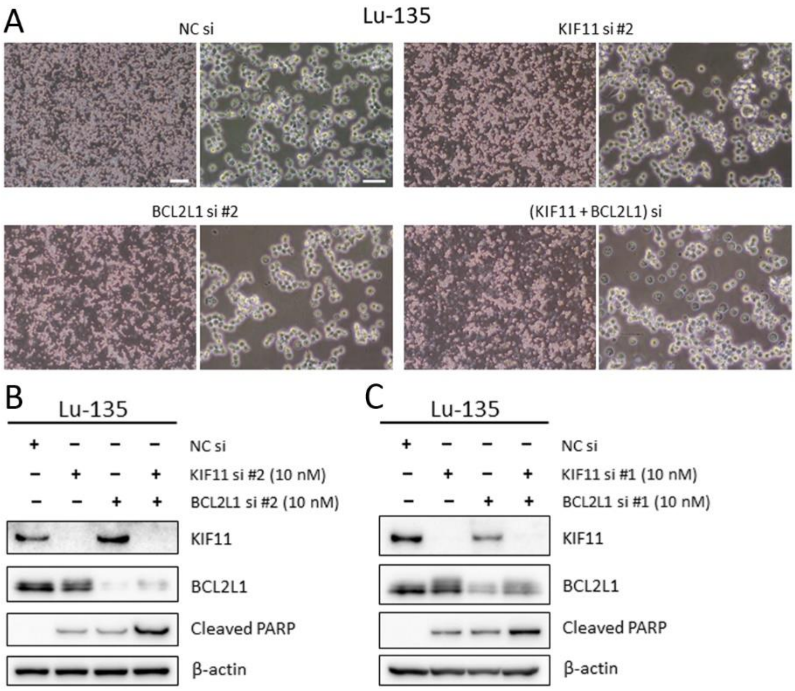


**Supplementary Figure S5.** Small interfering RNA (siRNA)-mediated knockdown of both KIF11 and BCL2L1 induces high levels of apoptosis in Lu-135 cells. (A) Phase contrast images of Lu-135 cells with KIF11 knockdown alone, BCL2L1 knockdown alone, dual knockdown of KIF11 and BCL2L1, or controls. Cells reverse transfected with the indicated siRNAs (10 nM each) were cultured for 48 hours. Scale bars = 200 µm (left; low magnification) and 50 µm (right; high magnification). (B) Western blot analysis of the cells treated as described in (A). (C) Western blot analysis of the treated Lu-135 cells. Cells reverse transfected with the indicated siRNAs (10 nM each) were cultured for 48 hours.

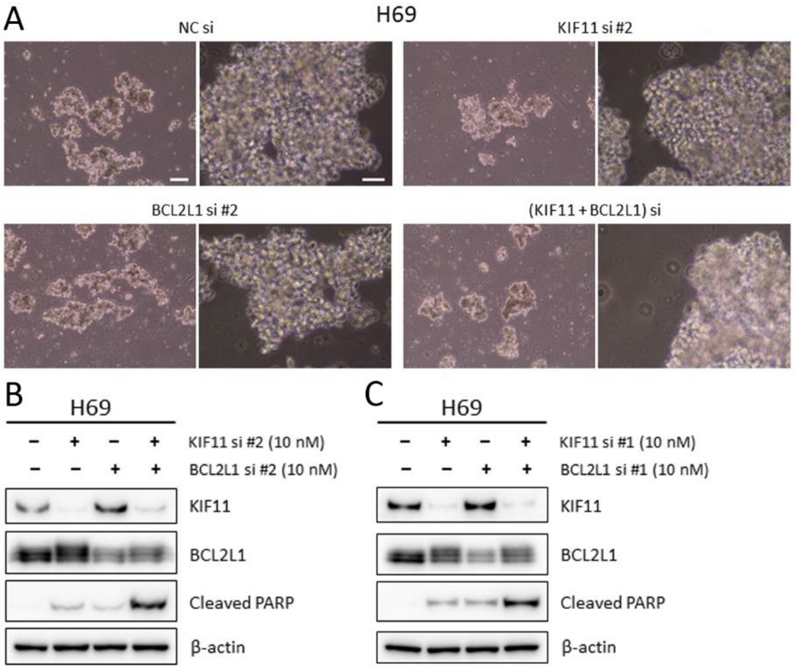


**Supplementary Figure S6.** Small interfering RNA (siRNA)-mediated knockdown of both KIF11 and BCL2L1 induces high levels of apoptosis in H69 cells. (A) Phase contrast images of H69 cells with KIF11 knockdown alone, BCL2L1 knockdown alone, dual knockdown of KIF11 and BCL2L1, or controls. Cells reverse transfected with the indicated siRNAs (10 nM each) were cultured for 48 hours. Scale bars = 200 µm (left; low magnification) and 50 µm (right; high magnification). (B) Western blot analysis of the cells treated as described in (A). (C) Western blot analysis of the treated H69 cells. Cells reverse transfected with the indicated siRNAs (10 nM each) were cultured for 48 hours.
